# Multi-Sensor Detection with Particle Swarm Optimization for Time-Frequency Coded Cooperative WSNs Based on MC-CDMA for Underground Coal Mines

**DOI:** 10.3390/s150921134

**Published:** 2015-08-27

**Authors:** Jingjing Xu, Wei Yang, Linyuan Zhang, Ruisong Han, Xiaotao Shao

**Affiliations:** 1 School of Electronic and Information Engineering, Beijing Jiaotong University, Beijing 100044, China; E-Mails: xujingjing@bjtu.edu.cn (J.X.); ruisong@bjtu.edu.cn (R.H.); xtshao@bjtu.edu.cn (X.S.); 2 Institute of Acoustics, Academia Sinica, Beijing 100080, China; E-Mail: happyzhangly@126.com

**Keywords:** underground coal mine, multicarrier code division multiple access (MC-CDMA), coded cooperation, multi-sensor detection, power allocation, particle swarm optimization

## Abstract

In this paper, a wireless sensor network (WSN) technology adapted to underground channel conditions is developed, which has important theoretical and practical value for safety monitoring in underground coal mines. According to the characteristics that the space, time and frequency resources of underground tunnel are open, it is proposed to constitute wireless sensor nodes based on multicarrier code division multiple access (MC-CDMA) to make full use of these resources. To improve the wireless transmission performance of source sensor nodes, it is also proposed to utilize cooperative sensors with good channel conditions from the sink node to assist source sensors with poor channel conditions. Moreover, the total power of the source sensor and its cooperative sensors is allocated on the basis of their channel conditions to increase the energy efficiency of the WSN. To solve the problem that multiple access interference (MAI) arises when multiple source sensors transmit monitoring information simultaneously, a kind of multi-sensor detection (MSD) algorithm with particle swarm optimization (PSO), namely D-PSO, is proposed for the time-frequency coded cooperative MC-CDMA WSN. Simulation results show that the average bit error rate (BER) performance of the proposed WSN in an underground coal mine is improved significantly by using wireless sensor nodes based on MC-CDMA, adopting time-frequency coded cooperative transmission and D-PSO algorithm with particle swarm optimization.

## 1. Introduction

There is a high demand for multimedia monitoring such as video and environmental parameters in underground coal mine production operations [1,2,3,4]. However, the existing wired monitoring systems cannot meet the requirements of safe production in underground coal mines, since there are a large number of blind monitoring areas. An underground wireless sensor network (WSN) can be an important supplement to a wired monitoring system due to its characteristics of flexible and rapid deployment. Therefore, the development of wireless sensor network (WSN) technology which is adaptive to underground channel conditions has an important theoretical and practical value for safety monitoring in underground coal mines.

Compared with wireless channels on the ground, underground wireless channels experience much more serious multipath fading along confined tunnels and the transmission conditions are much worse [5]. In underground coal mines, there are numerous challenges for applying WSNs such as long distances and unreliable data transmission [6]. The transmission rate of the WSN based on ZigBee is low, and it cannot satisfy the requirements for multimedia monitoring of an underground coal mine. Besides, with ZigBee the transmission distances are short and the reliability is low in underground coal mines [7,8]. Different from the wireless communication on the ground, the space, time and frequency resources in an underground coal mine are open, and they can be utilized efficiently for wireless transmission based on MC-CDMA to improve the quality of wireless transmission in underground coal mine tunnel [9].

The confined space of underground tunnel appears to be a banded structure [1,2,3]. Due to the different transmission distances to the sink node, it turns out that the channel conditions are better for some wireless sensor nodes while they are poorer for other wireless sensor nodes. A cooperative transmission strategy in which sensors with good channel conditions assist sensors with poor channel conditions is proposed to achieve space diversity gain which will improve the overall wireless transmission performance [10,11]. Therefore, a WSN based on the time-frequency coded cooperative MC-CDMA method is constructed for underground coal mines to improve the wireless transmission performance and reliability.

The channel fading problem is different for sensors at different locations. Therefore, the orthogonality between the MC-CDMA subcarriers is damaged to some extent, which will lead to MAI and influence the BER performance of the WSN. In order to solve this problem, multi-sensor detection (MSD) is adopted to improve the BER performance of the WSN for underground coal mines. Optimal multi-sensor detection (O-MSD) requires the detection of all possible bit sequences at the receiving sensor to find the best sequence that maximizes the objective function [12,13,14]. Theoretically, the O-MSD detection performance is optimal. However, the complexity of O-MSD increases exponentially with the number of sensors, which is contrary to the energy saving requirements of wireless sensors. Decorrelating multi-sensor detection (D-MSD) is a suboptimal detection approach where the receiving sensor demodulates the sending message of each transmitting sensor by using the channel state information (CSI) and spreading codes of transmitting sensors [15]. The complexity of D-MSD is lower than that of O-MSD, but its BER is much higher than that of O-MSD [16,17].

In order to make the detection performance of the time-frequency coded cooperative MC-CDMA WSN close to that O-MSD with relatively low complexity, a kind of multi-sensor detection with particle swarm optimization based on D-MSD (D-PSO) was proposed which optimizes the output of D-MSD at cooperative sensors and sink nodes with particle swarm optimization [18,19,20]. With this method, the BER performance of the proposed WSN for underground coal mines is improved significantly with relatively low complexity.

The rest of this paper is arranged as follows: the multi-sensor detection model of the time-frequency coded cooperative MC-CDMA WSN for underground coal mines is set up in Section 2. The transmitting and receiving signals of two phases for the proposed coded cooperative MC-CDMA WSN are analyzed in Section 3. The D-PSO algorithm for the proposed WSN is introduced and discussed in Section 4. Simulation results are presented to verify the performance of the proposed algorithm in Section 5. Finally, the conclusions of this paper are summarized in Section 6.

## 2. System Model

**Figure 1:**
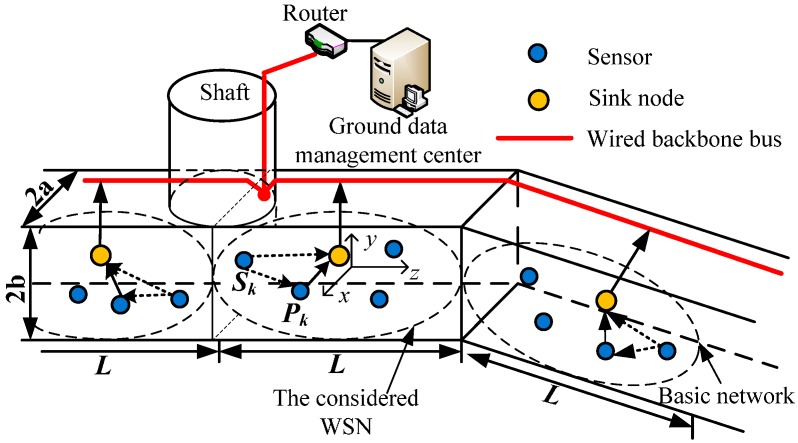
The system model.

Figure 1 shows the architecture of the time-frequency coded cooperative MC-CDMA for an underground coal mine. The network is composed of three layers, *i.e.*, the WSNs deployed in the underground coal mine, the wired backbone bus and the monitoring center on the ground [21,22]. As shown in Figure 1, the WSN consists of sink nodes and different kinds of wireless sensor nodes to form a banded coverage area along mine tunnels which monitor the multimedia information such as video and environment parameters in the underground coal mine. The varied monitoring information is transmitted to the sink node that connects with the wired backbone bus. The ground monitoring center receives the monitoring information through the wired backbone bus to realize comprehensive monitoring for the underground coal mine.

Assume that the width and height of mine tunnel shown in Figure 1 are 2a and 2b respectively and the coverage distance of each WSN is about *L*. Sink nodes are located in the central section of the considered WSN. A Cartesian coordinate system is set up with the sink node as reference point [23].

### 2.1. Coded Cooperative WSN Based on MC-CDMA

**Figure 2:**
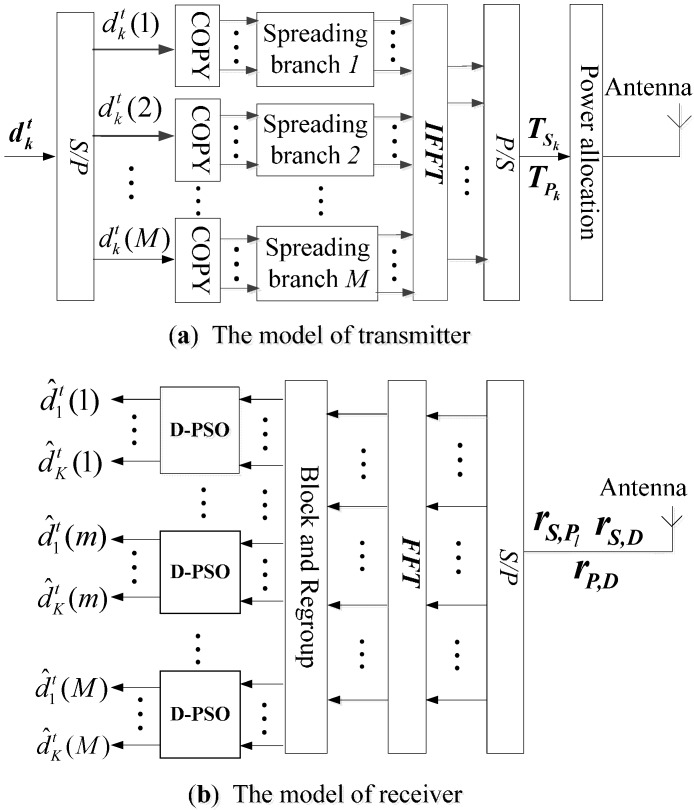
Proposed transmitter and receiver based on MC-CDMA.

It can be seen from Figure 1 that the distance to the sink node is different for sensors with different positions in the considered WSN. The channel conditions of sensors that are close to the sink node, such as $P_{1}$, are usually better; on the contrary, the channel conditions of sensors that are far from the sink node, such as $S_{1}$, are usually poor. In order to improve the wireless transmission performance of sensors with poor channel conditions, the sensors whose average channel gain exceeds a threshold η are elected to be the alternative group of cooperative sensors. *K* source sensors with poor channel conditions select their cooperative sensors from the alternative group, respectively, to form *K* pairs of partner sensors. For example, the source sensor $S_{1}$ and cooperative sensor $P_{1}$ form a pair of partner sensors as shown in Figure 1 [24]. Figure 2a,b show the block diagrams of the transmitter and receiver of sensor nodes based on MC-CDMA in an underground coal mine.

**Figure 3:**
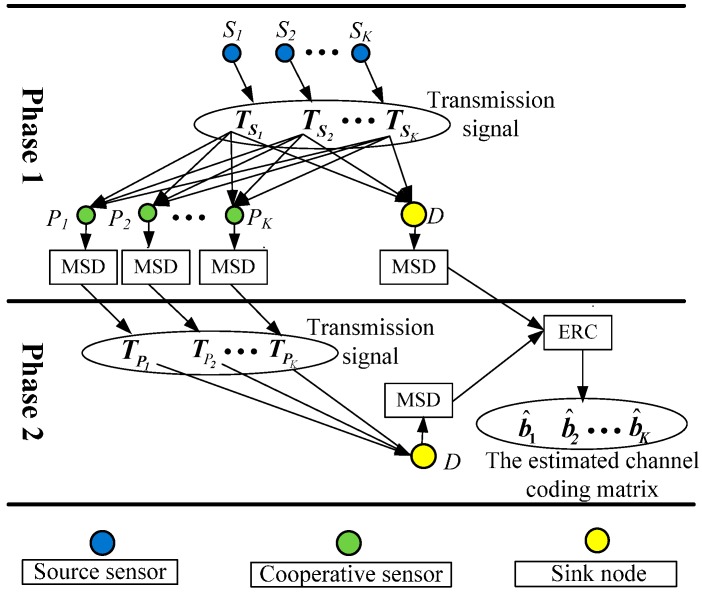
The block diagram of signal transmission in two phases: *t* = 1: Phase 1; *t* = 2: Phase 2.

Figure 3 illustrates the time-frequency coded cooperative transmission of sensors in two phases. In phase 1, signals $T_{S_{1}},T_{S_{2}},\cdots ,T_{S_{K}}$ are transmitted by source sensors $S_{1},S_{2},\cdots ,S_{K}$ after their time-frequency coding frames are modulated by MC-CDMA. The signals in phase 1 received by the cooperative sensors and sink node are processed by MC-CDMA demodulation, multi-sensor detection and time-frequency decoding. In phases 2, cooperative sensors assist source sensors to transmit signals. Signals $T_{P_{1}},T_{P_{2}},\cdots ,T_{P_{K}}$ are transmitted by cooperative sensors after the detected original information of source sensors is recoded and modulated by MC-CDMA. The signals in phase 2 received by the sink node are processed by MC-CDMA demodulation, multi-sensor detection and time-frequency decoding. Finally, the channel coding frames detected in phase 1 and phase 2 are combined by the sink node to obtain the estimated channel coding frames of the *K* source sensors.

### 2.2. Channel Model

A waveguide model with multimode is applied to the channel model of an underground tunnel. The attenuation and phase shift coefficient of each mode can be described as [25]:

(1) αmn=1a(mπ2ak)2Re(kv¯kv¯−1)+1b(nπ2bk)2Re(1kh¯−1)

(2) $$\beta_{mn}=\sqrt{k^{2}-{\left(\frac{m\pi }{2a}\right)}^{2}-{\left(\frac{n\pi }{2b}\right)}^{2}}$$

where Re(·) represents the real part of the results. (m,n) is the mode order, which indicates the reflection number of electromagnetic wave from vertical and horizontal walls of mine tunnel. The more reflection number (m,n) is, the higher the mode order gets. ${\bar{k}}_{h}$ and ${\bar{k}}_{v}$ are the electrical parameters of vertical and horizontal walls respectively: $\bar{k_{h}}=\frac{\varepsilon_{0}\varepsilon_{h}+\frac{\sigma_{h}}{j2\pi f_{n_{c}}}}{\varepsilon_{0}\varepsilon_{a}+\frac{\sigma_{a}}{j2\pi f_{n_{c}}}}$, $\bar{k_{v}}=\frac{\varepsilon_{0}\varepsilon_{v}+\frac{\sigma_{v}}{j2\pi f_{n_{c}}}}{\varepsilon_{0}\varepsilon_{v}+\frac{\sigma_{a}}{j2\pi f_{n_{c}}}}$. The wave number for the considered WSN is defined as $k=2\pi f_{n_{c}}\sqrt{\mu_{0}\varepsilon_{0}\varepsilon_{a}}$, where $\varepsilon_{0}$ is the permittivity of vacuum space; $\mu_{0}$ is the permeability which is same for the air, vertical and horizontal walls. $\varepsilon_{v}$, $\varepsilon_{h}$, $\varepsilon_{a}$ and $\sigma_{v}$, $\sigma_{h}$, $\sigma_{a}$ are the relative permittivity and conductivity of vertical, horizontal walls and the air in underground tunnel respectively. $f_{n_{c}}$ is the central frequency of *n_c_-*th subcarrier of wireless sensor nodes based on MC-CDMA.

Assume that the coordinates of a wireless sensor in the considered WSN are (*x*, *y*, *z*) as shown in Figure 1. Therefore, the field intensity of the sensor with distribution mode (m,n) is given by

(3) $$E_{m,n}^{eign}(x,y)\underline{\sim }\sin(\frac{m\pi }{2a}x+\phi_{x})\cdot \cos(\frac{n\pi }{2b}y+\phi_{y})$$

where φ*_x_* = 0 if *m* is even; φ*_x_* = π/2 if *m* is odd; φ*_y_* = π/2 if *n* is even; φ*_y_* = 0 if *n* is odd.

The channel gain of *n_c_*th subcarrier between transmitting sensor *i*(*x*_0_, *y*_0_, *z*_0_) and receiving sensor *j*(*x*, *y*, *z*) in the considered WSN is expressed as $h_{i,j}^{n_{c}}$. The channel gain of each subcarrier is obtained by adding up the front 50 modes which are stronger. Thus, the channel gain of *n_c_-*th subcarrier between sensor *i* and sensor *j* is given by:

(4) $$h_{i,j}^{n_{c}}=\sqrt{G_{t}G_{r}}\sum_{(m,n)\in Nmode}C_{mn}(x_{0},y_{0})E_{m,n}^{eign}\text{(}x,y\text{)}e^{-\text{(}\alpha_{mn}+j\beta_{mn}\text{)}\cdot \left|z-z_{0}\right|}$$

where, *C_mn_* is the mode intensity corresponding to mode (m,n) of the sensor, which is given by $C_{mn}(x,y)=\frac{\pi }{ab\sqrt{1-{\left(\frac{m\pi }{2ak}\right)}^{2}-{\left(\frac{n\pi }{2bk}\right)}^{2}}}E_{m,n}^{eign}(x,y)$; *G_t_* and *G_r_* are the antenna gains of transmitting sensor and receiving sensor respectively; *N_mode_* is a collection of the 50 modes.

It can be seen from Equation (4) that the channel gain of each subcarrier depends on its coordinates in the considered WSN when the *G_t_* and *G_r_* are invariant. Thus, the channel gain matrix $h_{i,j}$ between transmitting sensor *i*(*x_0_, y_0_, z_0_*) and receiving sensor *j*(*x*, *y*, *z*) can be expressed as:

(5) $$h_{i,j}=diag(h_{i,j}(1),h_{i,j}(2),\cdots ,h_{i,j}(N_{c}))\in (N_{c}\times N_{c})$$

## 3. Time-Frequency Coded Cooperative Transmission Based on MC-CDMA and Power Allocation

Assume that there are *K* source sensors which send messages to the sink node simultaneously in the considered WSN shown in Figure 1. The spreading code of source sensor $S_{k}$ and its cooperative sensor $P_{k}$ is expressed as $c_{k}={\left[c_{k}(1),\cdots ,c_{k}(g),\cdots ,c_{k}(G)\right]}^{T}$ with spreading gain *G* [26]. Thus, the *G × K* spreading code matrix c of *K* source sensors or *K* cooperative sensors is given by $c_{G\times K}=[c_{1},\cdots ,c_{k},\cdots ,c_{K}]$. Moreover, the spreading codes between wireless sensors are orthogonal, that is $\frac{1}{G}[c^{T}c]=I$, where $c^{T}$ is the transposed matrix of c, and I is the unit matrix.

### 3.1. Time-Frequency Coded Cooperative Transmission of Source Sensors and Cooperative Sensors in Two Phases

**Figure 4:**
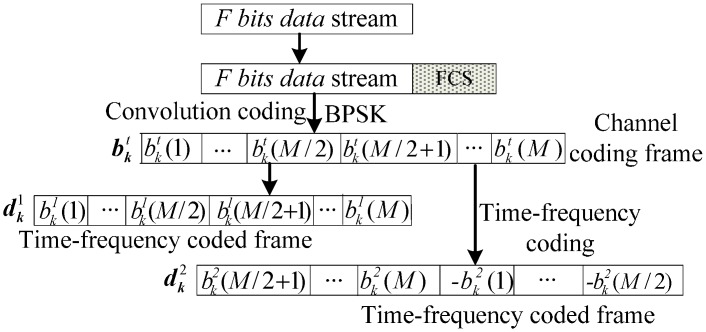
Time-frequency coding of the original data stream. *t* = 1: Phase 1; *t* = 2: Phase 2.

Before the signals are transmitted by *K* source sensors in the considered WSN, Frame Check Sequence (FCS) should be added to the original data stream firstly, then convolution coding and BPSK modulating are applied to obtain the *M* bits channel coding frame $b_{k}^{t}$. Then, the channel coding frame $b_{k}^{t}$ will be coded according to Figure 4 to get the time-frequency coded frame $d_{k}^{1}$ and $d_{k}^{2}$ which are corresponding to phase 1 and phase 2, respectively.

#### 3.1.1. Phase 1

In phase 1, each time-frequency coding frame $d_{k}^{1}$ of source sensor $S_{k}$ is transmitted after MC-CDMA modulation. The transmitting signal vector is given by:

(6) $$\begin{array}{l} T_{S_{k}}=[\underset{G}{\underset{︸}{c_{k}(1)d_{k}^{1}(1)e^{j2\pi f_{1}},\cdots ,c_{k}(G)d_{k}^{1}(1)e^{j2\pi f_{G}}}},\cdots ,\underset{G}{\underset{︸}{c_{k}(1)d_{k}^{1}(M)e^{j2\pi f_{(M-1)\times G}},\cdots ,c_{k}(G)d_{k}^{1}(M)e^{j2\pi f_{M\times G}}}}]\\ \in (1\times Nc) \end{array}$$

Therefore, the received signals at cooperative sensor $P_{l}(l=1,\cdots ,K)$ and the sink node D from *K* source sensors respectively are [27]:

(7) $$r_{S,P_{l}}=\sum_{k=1}^{K}\sqrt{E_{S_{k}}}\times T_{S_{k}}\times h_{S_{k},P_{l}}+N$$

(8) $$r_{S,D}=\sum_{k=1}^{K}\sqrt{E_{S_{k}}}\times T_{S_{k}}\times h_{S_{k},D}+N$$

where $E_{S_{k}}$ is the average transmit power of source sensor $S_{k}$; $h_{S_{k},P_{l}}$ and $h_{S_{k},D}$ are the channel gain matrix between source sensor $S_{k}$ and cooperative sensor $P_{l}$ as well as between source sensor $S_{k}$ and the sink node D; N is the noise vector of channel between sensors, $N=[n_{1},n_{2},\cdots ,n_{Nc}]\in 1\times Nc$. Without loss of generality, the channel noise of each narrow subcarrier is assumed to be additive white Gaussian noise (AWGN) with zero-mean and σ*^2^*-covariance.

#### 3.1.2. Phase 2

In phase 2, cooperative sensor $P_{k}$ recodes the data stream of source sensor $S_{k}$ detected according to Figure 4 to get the time-frequency coded frame $d_{k}^{2}$. Then $d_{k}^{2}$ will be retransmitted to the sink node after MC-CDMA modulation illustrated as in Figure 2a. The transmitting signal vector is given by:

(9) $$\begin{array}{l} T_{P_{k}}=[\underset{G}{\underset{︸}{c_{k}(1)d_{k}^{2}(1)e^{j2\pi f_{1}},\cdots ,c_{k}(G)d_{k}^{2}(1)e^{j2\pi f_{G}}}},\cdots ,\underset{G}{\underset{︸}{c_{k}(1)d_{k}^{2}(M)e^{j2\pi f_{(M-1)\times G}},\cdots ,c_{k}(G)d_{k}^{2}(M)e^{j2\pi f_{M\times G}}}}]\\ \in (1\times Nc) \end{array}$$

Therefore, the received signal vector from *K* cooperative sensors at the sink node is:

(10) $$r_{P,D}=\sum_{k=1}^{K}\sqrt{E_{P_{k}}}\times T_{P_{k}}\times h_{P_{k},D}+N$$

where, $E_{P_{k}}$ is the average transmit power of cooperative sensor $P_{k}$; $h_{P_{k},D}$ is the channel gain matrix between cooperative sensor $P_{k}$ and the sink node D.

### 3.2. Power Allocation between Source Sensor and Its Cooperative Sensor

Generally, the battery energy of a wireless sensor is limited [28,29,30]. It is therefore beneficial for the performance of the WSN to allocate node power reasonably. Therefore, a power allocation algorithm is proposed to distribute the power to source sensor and cooperative sensor according to their channel conditions to the sink node. The total transmission power of a pair of partner sensors is assumed to be *E*, *i.e.*, $E_{S_{k}}+E_{P_{k}}=E$. Then, the transmitted power of source sensor $S_{k}$ and its cooperative sensor $P_{k}$ are distributed according to their channel conditions to the sink node D, which are formulated as:

(11) $$E_{S_{k}}=\frac{{\bar{h}}_{P_{k},D}}{{\bar{h}}_{S_{k},D}+{\bar{h}}_{P_{k},D}}E$$

(12) $$E_{P_{k}}=\frac{{\bar{h}}_{S_{k},D}}{{\bar{h}}_{S_{k},D}+{\bar{h}}_{P_{k},D}}E$$

where ${\bar{h}}_{S_{k},D}$ is the average channel gain between source sensor $S_{k}$ and sink node D, ${\bar{h}}_{S_{k},D}=\frac{1}{N_{c}}\sum_{n_{c}=1}^{n_{c}=N_{c}}h_{S_{k},D}(n_{c})$; ${\bar{h}}_{P_{k},D}$ is the average channel gain between cooperative sensor $P_{k}$ and sink node D, ${\bar{h}}_{P_{k},D}=\frac{1}{N_{c}}\sum_{n_{c}=1}^{n_{c}=N_{c}}h_{P_{k},D}(n_{c})$.

With the proposed power allocation scheme, the power distributed between a pair of partner sensors is balanced according to their channel states. Thus, the situation that a wireless sensor with poor channel condition is allocated insufficient power which may result in a higher BER can be avoided.

## 4. Multi-Sensor Detection with Particle Swarm Optimization

Due to the different fading for different sensors, the orthogonality between the subcarriers of receiving signals $r_{S,P_{l}}$, $r_{S,D}$ and $r_{P,D}$ shown in Equations (7), (8) and (10) is damaged to some extent, which will lead to MAI. To reduce the influence of MAI and improve the BER performance of the WSN for underground coal mines, a D-PSO algorithm with particle swarm optimization at cooperative sensors and sink node was adopted.

### 4.1. Decorrelation Detection with Particle Swarm Optimization at Cooperative Sensor

As shown in Figure 3, the cooperative sensors $P_{l}(l=1,\cdots ,K)$ receive signals from *K* source sensors in phase 1. In order to assist its source sensor $S_{l}(l=1,\cdots ,K)$, cooperative sensor $P_{l}$ detects the original data stream of source sensor $S_{l}$ with multi-sensor detection (MSD). As depicted in Figure 2b, the signal $r_{S,P_{l}}$ received at cooperative sensor $P_{l}$ is serial-to-parallel converted into a *N_c_*-path parallel signals. Then, the *N_c_*-path parallel signals are turned into frequency-domain signals $y_{S,P_{l}}$ by Fast Fourier Transform (FFT), which is given by:

(13) $$\begin{array}{cc} y_{S,P_{l}} & ={\left[y_{S,P_{l}}(1),y_{S,P_{l}}(2),\cdots ,y_{S,P_{l}}(N_{c})\right]}^{T}\\ & =\left[\begin{array}{c} \sum_{k=1}^{K}\sqrt{E_{S_{k}}}c_{k}(1)d_{k}^{1}(1)h_{S_{k},P_{l}}(1)+n_{1}e^{-j2\pi f_{1}}\\ \sum_{k=1}^{K}\sqrt{E_{S_{k}}}c_{k}(2)d_{k}^{1}(1)h_{S_{k},P_{l}}(2)+n_{2}e^{-j2\pi f_{2}}\\ \vdots \\ \sum_{k=1}^{K}\sqrt{E_{S_{k}}}c_{k}(G)d_{k}^{1}(M)h_{S_{k},P_{l}}(Nc)+n_{N\text{c}}e^{-j2\pi f_{N_{c}}} \end{array}\right] \end{array}$$

Assume that the *m-*th symbols of all time-frequency coding frames of *K* source sensors form the symbol vector $d_{S,P_{l}}^{m}$ which can be expressed as $d_{S,P_{l}}^{m}={\left[d_{1}^{1}(m),\cdots ,d_{k}^{1}(m),\cdots ,d_{K}^{1}(m)\right]}^{T}$. The detection vector of $d_{S,P_{l}}^{m}$ is given by:

(14) $$\begin{array}{cc} y_{S,P_{l}}^{m} & ={\left[y_{S,P_{l}}^{m}(1),y_{S,P_{l}}^{m}(2),\cdots ,y_{S,P_{l}}^{m}(G)\right]}^{T}\\ & ={\left[y_{S,P_{l}}((m-1)G+1),y_{S,P_{l}}((m-1)G+2),\cdots ,y_{S,P_{l}}(mG)\right]}^{T}\\ & =\left[\begin{array}{c} \sum_{k=1}^{K}\sqrt{E_{S_{k}}}c_{k}(1)d_{k}^{1}(m)h_{S_{k},P_{l}}((m-1)G+1)+n_{(m-1)G+1}e^{-j2\pi f_{(m-1)G+1}}\\ \vdots \\ \sum_{k=1}^{K}\sqrt{E_{S_{k}}}c_{k}(G)d_{k}^{1}(m)h_{S_{k},P_{l}}(mG)+n_{mG}e^{-j2\pi f_{mG}} \end{array}\right]m=1,\cdots ,M \end{array}$$

The cooperative sensor $P_{l}$ apply D-MSD to estimate the vector of $d_{S,P_{l}}^{m}$, which can be expressed as:

(15) $$\hat{d}\_D_{S,P_{l}}^{m}={\left[{\hat{d}}_{1}^{1}(m),\cdots ,{\hat{d}}_{k}^{1}(m),\cdots ,{\hat{d}}_{K}^{1}(m)\right]}^{T}=pinv(Q_{S,P_{l}}^{m})\times y_{S,P_{l}}^{m}$$

where pinv() represents the pseudo inverse matrix. Matrix $Q_{S,P_{l}}^{m}$ is defined as:

(16) $$Q_{S,P_{l}}^{m}=\left[\begin{array}{c} c_{1}(1)h_{S_{1},P_{l}}((m-1)G+1),\cdots ,c_{K}(1)h_{S_{K},P_{l}}((m-1)G+1)\\ c_{1}(2)h_{S_{1},P_{l}}((m-1)G+2),\cdots ,c_{K}(2)h_{S_{K},P_{l}}((m-1)G+2)\\ \vdots \\ c_{1}(G)h_{S_{1},P_{l}}(mG),\cdots ,c_{K}(G)h_{S_{K},P_{l}}(mG) \end{array}\right]$$

When the channel noise is ignored, $Q_{S,P_{l}}^{m}$ meets the equation: $y_{S,P_{l}}^{m}=Q_{S,P_{l}}^{m}d_{S,P_{l}}^{m}$ [31].

Particle swarm optimization (PSO) is a kind of heuristic, stochastic and iterative optimization search algorithm, which has the advantages of good convergence performance and simple implementation, *etc*. In phase 1, cooperative sensors use the D-PSO algorithm to iteratively update the output of D-MSD with particle swarm optimization [18,32]. Consequently, the *K* × *M* estimated matrix of time-frequency coded frames of *K* source sensors is obtained at each cooperative sensor. Assume that the iteration number is *Max_dt* and the particle swarm scale is *N_p_* for each detection. The position and velocity of *p-*th particle at *i*th iteration for *m-*th symbol vector in *K* dimensional space are $X_{p,i}^{m}={\left[X_{p1,i}^{m},X_{p2,i}^{m},\cdots ,X_{pk,i}^{m},\cdots ,X_{pK,i}^{m}\right]}^{T}$ and $V_{p,i}^{m}={\left[V_{p1,i}^{m},V_{p2,i}^{m},\cdots ,V_{pk,i}^{m},\cdots ,V_{pK,i}^{m}\right]}^{T}$ respectively. The cooperative sensor $P_{l}$ with D-PSO algorithm sets the output $\hat{d}\_D_{S,P_{l}}^{m}$ of D-MSD given by Equation (15) as the initial position of its first particle at the first iteration, *i.e.*, $X_{1,1}^{m}=\hat{d}\_D_{S,P_{l}}^{m}$.

The objective function for *m-*th symbol vector is applied by cooperative sensor $P_{l}$ to evaluate the position of *p-*th particle at *i-*th iteration:

(17) F(Xp,im)=Re[2(Xp,im)TAS(QS,Plm)TyS,Plm-(Xp,im)TAS(QS,Plm)TASQS,PlmXp,im]

where $A_{S}$ is the average amplitude matrix of the transmitting signals of *K* source sensors, $A_{S}=\mathrm{diag}(\sqrt{E_{S_{1}}},\cdots ,\sqrt{E_{S_{k}}},\cdots ,\sqrt{E_{S_{K}}})$; Re(•) represents the real part. The higher the value of objective function is, the better the position of particle is.

The position and velocity of *p-*th particle at *i-*th iteration in *k-*th dimension for *m-*th symbol vector at cooperative sensor $P_{l}$ are updated as follows:

(18) $$V_{pk,i+1}^{m}=w\times V_{pk,i}^{m}+c_{1}\times r_{1}\times (pbest_{pk,i}^{m}-X_{pk,i}^{m})+c_{2}\times r_{2}\times (gbest_{k,i}^{m}-X_{pk,i}^{m})$$

(19) $$X_{pk,i+1}^{m}=\{\begin{array}{c} 1,\frac{1}{1+\exp(-V_{pk,i+1}^{m})}\geq rand\\ \text{-}1,\frac{1}{1+\exp(-V_{pk,i+1}^{m})}<rand \end{array}$$

where *w* is inertia weight coefficient which is used to control the update speed of particles; *r_1_* and *r_2_* are random numbers between [0, 1]. *c_1_* and *c_2_* are learning factors which are applied to accelerate the particle to the best position of particle swarm; $V_{max}\leq V_{pk,i}^{m}\leq -V_{max}$, where $V_{max}$ is the maximum velocity of the particles; $pbest_{p,i}^{m}$ is the local best position, that is the best position of *p-*th particle at *i-*th iteration for *m-*th symbol vector; $gbest_{i}^{m}$ is the global best position, which denotes the optimal detection result at *i-*th iteration for *m-*th symbol vector. $pbest_{p,i}^{m}$ and $gbest_{i}^{m}$ are determined by:

(20) $$pbest_{p,i}^{m}\text{=}\max\left\{F(X_{p,1}^{m}),F(X_{p,2}^{m}),\cdots ,F(X_{p,i}^{m})\right\}$$

(21) $$gbest_{i}^{m}\text{=}\max\left\{F(pbest_{1,i}^{m}),F(pbest_{2,i}^{m}),\cdots ,F(pbest_{N_{p},i}^{m})\right\}$$

The detection result of *m-*th symbol vector is obtained by D-PSO algorithm after the iteration number arrives at *Max_dt*, which is given by:

(22) $$\hat{d}\_PSO_{S,P_{l}}^{m}={\left[{\hat{d}}_{1}(m),\cdots ,{\hat{d}}_{k}(m),\cdots ,{\hat{d}}_{K}(m)\right]}^{T}=gbest_{Max\_dt}^{m}$$

Therefore, the cooperative sensor $P_{l}$ can get the estimated K×M matrix for the *M* bits of time-frequency coded frames of *K* source sensors in phase 1, which is given by:

(23) $$\begin{array}{ll} \hat{d}\_PSO_{S,P_{l}} & ={\left[{\hat{d}}_{1}^{1},\cdots ,{\hat{d}}_{k}^{1},\cdots ,{\hat{d}}_{K}^{1}\right]}^{T}\\ & =[\hat{d}\_PSO_{S,P_{l}}^{1},\cdots ,\hat{d}\_PSO_{S,P_{l}}^{m},\cdots \hat{d}\_PSO_{S,P_{l}}^{M}]\\ & =\left[\begin{array}{ccccc} {\hat{d}}_{1}^{1}(1) & \cdots & {\hat{d}}_{1}^{1}(m) & \cdots & {\hat{d}}_{1}^{1}(M)\\ \vdots & & \vdots & & \vdots \\ {\hat{d}}_{k}^{1}(1) & \cdots & {\hat{d}}_{k}^{1}(m) & \cdots & {\hat{d}}_{k}^{1}(M)\\ \vdots & & \vdots & & \vdots \\ {\hat{d}}_{K}^{1}(1) & \cdots & {\hat{d}}_{K}^{1}(m) & \cdots & {\hat{d}}_{K}^{1}(M) \end{array}\right]k=1,\cdots ,K \end{array}$$

Thus, the estimated channel coded frame ${\hat{b}}_{l}^{1}$ of source sensor $S_{l}$ can be obtained at cooperative sensor $P_{l}$ by decoding the time-frequency coded frame ${\hat{d}}_{l}^{1}$ according to Figure 4. Based on the above analysis, the basic procedure of D-PSO algorithm for a cooperative sensor $P_{l}(l=1,\cdots ,K)$ of the time-frequency coded cooperative MC-CDMA WSN can be given by the following steps:

(1) Convert the received signal vector $r_{S,P_{l}}$ into frequency-domain signals $y_{S,P_{l}}$ through serial-to-parallel converting and Fast Fourier Transform (FFT), then block and regroup $y_{S,P_{l}}$ to get the detection vector $y_{S,P_{l}}^{m}$ of the *m*-th symbol;
(2) Apply D-MSD to $y_{S,P_{l}}^{m}$, and set its output $\hat{d}\_D_{S,P_{l}}^{m}$ as the initial position of the first particle of D-PSO algorithm, *i.e.*, $X_{1,1}^{m}=\hat{d}\_D_{S,P_{l}}^{m}$;
(3) Update the position and velocity of each particle of the *m*-th symbol vector according to Equations (18) and (19);
(4) Update the local best position $pbest_{p,i}^{m}$ and global best position $gbest_{i}^{m}$ of the *m*-th symbol vector with Equations (20) and (21);
(5) Renew the iteration number, *i* = *i* + 1 and return to step (3). When the iteration time arrives at *Max_dt*, D-PSO algorithm outputs the multi-sensor detection result $\hat{d}\_PSO_{S,P_{l}}^{m}$ with particle swarm optimization.

In the iterations of the D-PSO algorithm, each particle adjusts its position continuously according to the objective function to gradually converge to the global best position. Since the D-PSO algorithm utilizes the output of D-MSD to provide a good initialization for the particle swarm, particles can quickly converge to the global best position. Though the complexity of D-PSO algorithm with O (*N_p_* × *Max_dt*) is slightly higher than that of D-MSD algorithm with O (*K*), it is much lower than that of O-MSD algorithm with O (2*K*). However, the simulation results in section 5 show that the BER performance of D-PSO algorithm is much better than that of D-MSD algorithm.

### 4.2. Decorrelating Detection with Particle Swarm Optimization at the Sink Node

#### 4.2.1. Phase 1

Similarly, the received signal vector $r_{S,D}$ from *K* source sensors at sink node D shown as in Equation (8) is also detected with D-PSO algorithm in the same way as cooperative sensor $P_{l}$. Therefore, the sink node D can get the estimated matrix for the *M* bits of time-frequency coded frames of *K* source sensors in phase 1:

(24) $${\hat{d}}_{S,D}^{1}=\left[\begin{array}{ccccc} {\hat{d}}_{1}^{1}(1) & \cdots & {\hat{d}}_{1}^{1}(m) & \cdots & {\hat{d}}_{1}^{1}(M)\\ \vdots & & \vdots & & \vdots \\ {\hat{d}}_{k}^{1}(1) & \cdots & {\hat{d}}_{k}^{1}(m) & \cdots & {\hat{d}}_{k}^{1}(M)\\ \vdots & & \vdots & & \vdots \\ {\hat{d}}_{K}^{1}(1) & \cdots & {\hat{d}}_{K}^{1}(m) & \cdots & {\hat{d}}_{K}^{1}(M) \end{array}\right]$$

#### 4.2.2. Phase 2

Just like in phase 1, the received signal vector $r_{P,D}$ from *K* cooperative sensors at sink node D shown as in Equation (10) is also detected with the D-PSO algorithm in the same way as cooperative sensor $P_{l}$ in phase 2. The sink node D can get the estimated matrix for the *M* bits of time-frequency coded frames from *K* cooperative sensors in phase 2, which is expressed as:

(25) $${\hat{d}}_{P,D}^{2}=\left[\begin{array}{ccccc} {\hat{d}}_{1}^{2}(1) & \cdots & {\hat{d}}_{1}^{2}(m) & \cdots & {\hat{d}}_{1}^{2}(M)\\ \vdots & & \vdots & & \vdots \\ {\hat{d}}_{k}^{2}(1) & \cdots & {\hat{d}}_{k}^{2}(m) & \cdots & {\hat{d}}_{k}^{2}(M)\\ \vdots & & \vdots & & \vdots \\ {\hat{d}}_{K}^{2}(1) & \cdots & {\hat{d}}_{K}^{2}(m) & \cdots & {\hat{d}}_{K}^{2}(M) \end{array}\right]$$

### 4.3. Combining the Detected Results of Two Phases at the Sink Node

As illustrated in Figure 3, the estimated matrix ${\hat{b}}_{S,D}^{1}$ and ${\hat{b}}_{P,D}^{2}$ detected in phase 1 and phase 2 are combined at the sink node D to get the channel coded *K* × *M* matrix $\hat{b}$ for the *M* bits channel coded frames of *K* source sensors, which is given by:

(26) $$\hat{b}={\left[{\hat{b}}_{1},\cdots ,{\hat{b}}_{k},\cdots ,{\hat{b}}_{K}\right]}^{T}=\frac{{\hat{b}}_{S,D}^{1}+{\hat{b}}_{P,D}^{2}}{2}$$

where ${\hat{b}}_{S,D}^{1}$ and ${\hat{b}}_{P,D}^{2}$ are the channel coded matrix derived from ${\hat{d}}_{S,D}^{1}$ and ${\hat{d}}_{P,D}^{2}$ after time-frequency decoding, respectively. For our proposed time-frequency coding scheme: ${\hat{b}}_{S,D}^{1}={\hat{d}}_{S,D}^{1}$ and: ${\hat{b}}_{P,D}^{2}=\left[\begin{array}{ccccc} {\hat{d}}_{1}^{2}(-(M/2+1)) & \cdots & {\hat{d}}_{1}^{2}(-M) & \cdots & {\hat{d}}_{1}^{2}(M/2)\\ \vdots & & \vdots & & \vdots \\ {\hat{d}}_{k}^{2}(-(M/2+1)) & \cdots & {\hat{d}}_{k}^{2}(-M) & \cdots & {\hat{d}}_{k}^{2}(M/2)\\ \vdots & & \vdots & & \vdots \\ {\hat{d}}_{K}^{2}(-(M/2+1)) & \cdots & {\hat{d}}_{K}^{2}(-M) & \cdots & {\hat{d}}_{K}^{2}(M/2) \end{array}\right]$.

The monitoring information is transmitted to the ground monitoring center by sink node through the wired backbone bus to realize the comprehensive monitoring of the underground coal mine.

## 5. Results and Discussion

In this section, the average BER performance of the wireless sensor nodes for the time-frequency coded cooperative MC-CDMA WSN in uplinks is evaluated by Monte Carlo simulation, which is carried out via Matlab simulation on an IBM server with Linux environment at the Wireless Sensor Networks Laboratory of Beijing Jiaotong University. As shown in Figure 1, assume that the sink node in the considered WSN is located at coordinates (2.5, 1.5, 0) and the coordinates of a wireless sensor node are (2.5, −1.5, *z*), 0 < *z* ≤ 600. It is also assumed that the number of source sensors is equal to the spreading gain, *i.e.*, *K* = *G*. Meanwhile, the channel state information (CSI) between the transmitting and receiving sensors is available. The simulation parameters of the mine tunnel, time-frequency coding of wireless sensor node, MC-CDMA modulation and D-PSO algorithm are shown in Table 1, Table 2, Table 3 and Table 4, respectively. Since the channel gain is different for different subcarrier of MC-CDMA, it is more reasonable and simple to apply total input signal noise ratio (SNR) namely *E/*σ*^2^* to describe the BER performance [25,33].

**Table 1:** The simulation parameters of the mine tunnel.

Parameters	Mark	Value
Permittivity of vertical wall	$\varepsilon_{v}$	5
Permittivity of horizontal wall	$\varepsilon_{h}$	5
Permittivity of the air	$\varepsilon_{a}$	1
Conductivity of vertical wall	$\sigma_{v}$	0.01
Conductivity of horizontal wall	$\sigma_{h}$	0.01
Conductivity of the air	$\sigma_{a}$	0
Width of the mine tunnel	2 *a*	10 m
Height of the mine tunnel	2 *b*	6 m
Coverage length of WSN	*L*	1200 m
Antenna gain of transmitting sensor	*G_t_*	3 dB
Antenna gain of receiving sensor	*G_r_*	18 dB
Permittivity of vacuum space	$\varepsilon_{0}$	8.854 × 10^−12^
Permeability of the wall and air	$\mu_{0}$	1.256 × 10^−6^

**Table 2:** The simulation parameters of time-frequency coding.

Parameters	Mark	Value
Length of information bit stream	F = N_c_/G*R − n	29
Length of channel coding frame	*M* = N_c_/G*R	32
Length of CRC check bits (FCS)	*n*	3
Rate of convolutional coding	*R*	1/2
Modulation type	/	K

**Table 3:** The simulation parameters of MC-CDMA.

Parameters	Mark	Value
Center frequency	*f*_0_	900 MHz
Interval of the subcarriers	Δ*F*	15 kHz
Spreading code type	/	Walsh Hadamard

**Table 4:** The simulation parameters of D-PSO algorithm.

Parameters	Mark	Value
Learning factor	*c*_1_	2
Learning factor	*c*_2_	2
Inertia weight coefficient	*w*	0.99
Total number of particles	*N_p_*	20
Iteration time	*Max_dt*	30
Maximum velocity	*V_max_*	2

**Figure 5:**
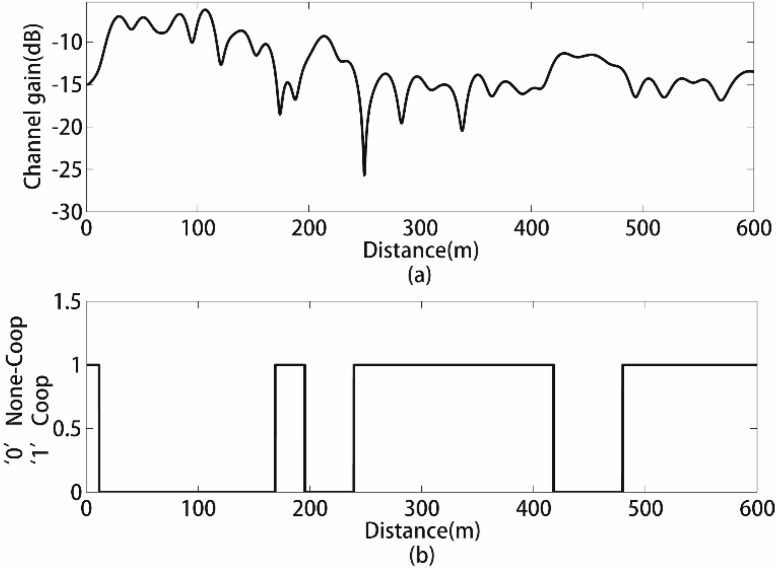
The average channel gain changes with the distance to the sink node in WSN.

The average channel gain between the wireless sensor node and the sink node of MC-CDMA that varies with the distance is shown in Figure 5a. It can be seen that the average channel gain presents a damped curve overall with some fluctuations.

In some areas of the considered WSN, the channel gain is relatively higher, such as when sensors are less than 100 m away from the sink node. However, the channel gain is relatively lower in some area, such as when sensors are more than 500 m away from the sink node. Assume that the channel gain threshold η is −13 dB. Consequently, the alternative cooperative sensors are distributed in the area where the channel gain is above the threshold of −13 dB. Figure 5b describes the transmission pattern of sensors according to the threshold η, where “0” represents none-cooperative transmission because of better channel conditions; “1” represents cooperative transmission because of the poor channel conditions. Assume that *K* source sensors are randomly distributed between 250 m–400 m and *J* alternative cooperative sensors are randomly distributed between 250 m–400 m and 20 m–180 m, *J* > *K*.

**Figure 6:**
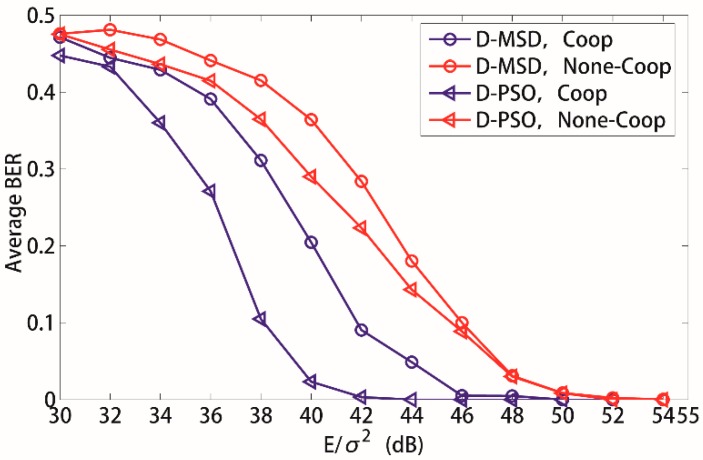
Influence of different transmission manner to the average BER. *K* = 8, *N_c_* = 256, $E_{S_{k}}=E_{P_{k}}$.

Figure 6 illustrates the average BER of source sensors *versus* total input SNR *E*/σ^2^ for the proposed WSN in an underground coal mine when the D-MSD algorithm and D-PSO algorithm are applied respectively. From Figure 6, it can be clearly observed that the average BER performance of the source sensors is improved significantly when the time-frequency coded cooperative transmission is applied both for D-MSD algorithm and D-PSO algorithm. For example, the time-frequency coded cooperative transmission outperforms none-cooperative transmission by 3 dB and 5 dB for the D-MSD algorithm and D-PSO algorithm, respectively, when the average BER is 0.25. From Figure 6, it can also be observed that the D-PSO algorithm outperforms the D-MSD algorithm by 3 dB for the time-frequency coded cooperative transmission when the average BER is 0.25.

**Figure 7:**
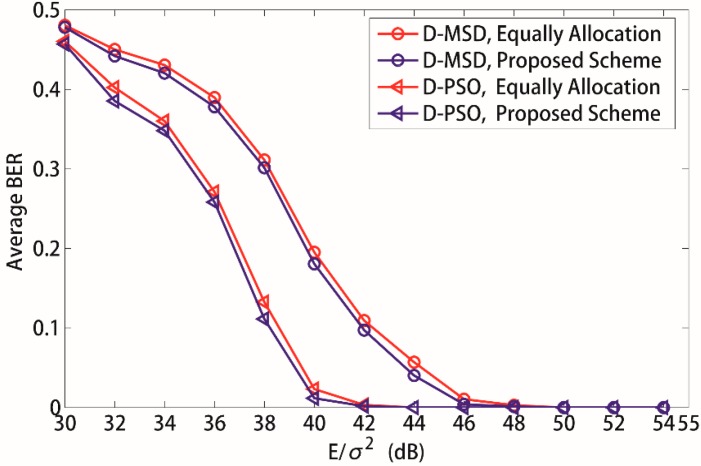
Influence of different power allocation scheme to the average BER. *K* = 8, *N_c_* = 256.

Figure 7 demonstrates the average BER of source sensors *versus* total input SNR *E/*σ*^2^* with equal and the proposed power allocation scheme when the D-MSD algorithm and D-PSO algorithm are applied to the time-frequency coded cooperative MC-CDMA WSN, respectively. From Figure 7, it can be observed that the average BER performance of source sensors is much better with the proposed power allocation scheme than that with equal power allocation scheme both for the D-MSD algorithm and D-PSO algorithm. This is because an equal power allocation scheme does not consider the channel conditions of the source sensor and its cooperative sensor. When the channel conditions from source sensor to the sink node are poor, the average BER of the source sensor will increase since the transmission power of the source sensor has not been correspondingly increased. Consequently, the overall performance of the proposed WSN will definitely be influenced. Therefore, the limited energy of wireless sensors based on MC-CDMA can be fully made use of with the proposed power allocation scheme to improve the performance of the WSN.

**Figure 8:**
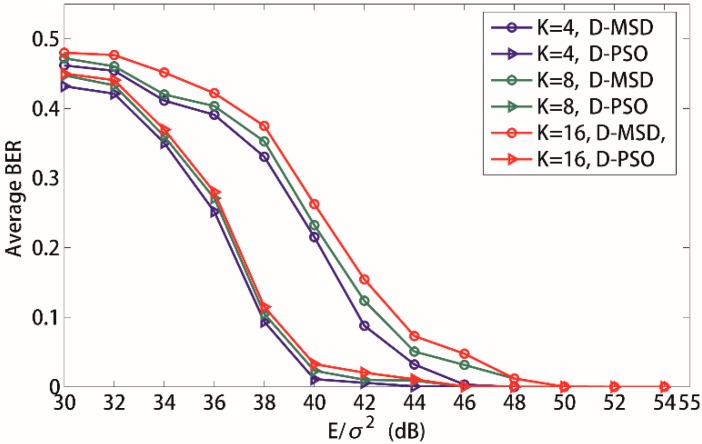
Influence of the number of source sensors to the average BER. *K* = 4, *N_c_* = 128; *K* = 8, *N_c_* = 256; *K* = 16, *N_c_* = 512.

Figure 8 depicts the average BER *versus* total input SNR *E/*σ*^2^* when the number of source sensors is 4, 8, and 16, respectively. Here, both the D-MSD algorithm and D-PSO algorithm are applied to the time-frequency coded cooperative MC-CDMA WSN. From Figure 8, it can be observed that the average BER of source sensors increases when the number of source sensors increases. This is because the MAI increases when the number of source sensors increases. For example, when the number of source sensors *K* varies from 4 to 16, the average BER of source sensors increases about 0.05 for 38 dB with the D-MSD algorithm. However, the average BER of source sensors increases only 0.02 for D-PSO algorithm at the same conditions. Obviously, it will benefit a lot for the average BER of source sensors to apply the D-PSO algorithm to the time-frequency coded cooperative MC-CDMA WSN in underground coal mines.

## 6. Conclusions

In order to improve the wireless transmission performance of a WSN in an underground coal mine, a WSN based on time-frequency coded cooperative MC-CDMA for underground coal mines is proposed:

(1) Wireless sensor nodes with MC-CDMA wireless transmission can make full use of the open space, time and frequency resources of the underground coal mine to improve the wireless transmission performance.
(2) Wireless sensor nodes with time-frequency coded cooperative wireless transmission can obtain coding gain and spatial diversity gain which will significantly improve the wireless transmission performance of sensors with bad channel conditions.
(3) The MAI among sensors of the time-frequency coded cooperative MC-CDMA WSN can be reduced effectively by adopting the D-PSO algorithm with particle swarm optimization which will also significantly improve the wireless transmission performance of the proposed WSN.

## References

[1] Zhang Y., Yang W., Han D., Kim Y.I. (2014). An Integrated Environment Monitoring System for Underground Coal Mines—Wireless Sensor Network Subsystem with Multi-Parameter Monitoring. Sensors.

[2] Han G., Zhang W., Zhang Y.P. (2009). An Experiment Study of the Propagation of Radio Waves in a Scaled Model of Long-Wall Coal Mining Tunnels. IEEE Antennas Wirel. Propag. Lett..

[3] Li J., Whisner B., Waynert J.A. (2013). Measurements of Medium-Frequency Propagation Characteristics of a Transmission Line in an Underground Coal Mine. IEEE Trans. Ind. Appl..

[4] Akyildiz I.F., Melodia T., Chowdhury K.R. (2008). Wireless Multimedia Sensor Networks: Applications and Testbeds. IEEE Proc..

[5] Sun Z., Akyildiz I.F., Hancke G.P. (2011). Dynamic Connectivity in Wireless Underground Sensor Networks. IEEE Commun. Soc..

[6] Zhang Y.P., Zheng G.X., Sheng J.H. (2001). Radio propagation at 900 MHz in underground coal mines. IEEE Trans. Antennas Propag..

[7] Byun J.S., Jeon B., Noh J., Kim Y., Park S. (2012). An intelligent self-adjusting sensor for smart home services based on ZigBee communications. IEEE Trans. Consum. Electron..

[8] Shrestha P.L., Hempel M., Sharif H., Chen H.-H. (2013). Modeling Latency and Reliability of Hybrid Technology Networking. IEEE Sens. J..

[9] Yang W., Cheng S., Sun J. (2000). The scheme study of the application of W-CDMA spread spectrum communication in mine mobile communication. J. Coal Sci. Eng. (China).

[10] Buzzi S., Saturnino D. (2010). A Game-Theoretic Approach to Energy-Efficient Power Control and Receiver Design in Cognitive CDMA Wireless Networks. IEEE Signal Process. Soc..

[11] Permuter H.H., Shamai S., Somekh-Baruch A. (2011). Message and State Cooperation in Multiple Access Channels. IEEE Trans. Inf. Theory.

[12] Botsinis P., Ng S.X., Hanzo L. (2013). Quantum Search Algorithms, Quantum Wireless, and a Low-Complexity Maximum Likelihood Iterative Quantum Multi-User Detector Design. IEEE Access.

[13] Zhang J., Chen S., Mu X., Hanzo L. (2012). Turbo Multi-User Detection for OFDM/SDMA Systems Relying on Differential Evolution Aided Iterative Channel Estimation. IEEE Trans. Commun..

[14] Zhao N., Wu Z., Zhao Y., Quan T. (2010). A Population Declining Mutated Ant Colony Optimization Multiuser Detector for MC-CDMA. IEEE Commun. Lett..

[15] Guo D. (2006). Performance of multicarrier CDMA in frequency-selective fading via statistical physics. IEEE Inf. Theory Soc..

[16] Yang L., Soo K.-K., Siu Y.-M., Chen R.-S. (2008). Hybrid Reduced-Complexity Multiuser Detector for CDMA Communication Systems. IEEE Trans. Veh. Technol..

[17] Nguyen G.D., Kompella S., Kam C. (2014). Achievable Throughput under BER Constraints via Transmission Scheduling and Multiuser Detection. IEEE Trans. Wirel. Commun..

[18] Soo K.K., Siu Y.M., Chan W.S. (2007). Particle Swarm Optimization Based Multiuser Detector for CDMA Communications. IEEE Veh. Technol. Soc..

[19] Liu H., Li J. (2008). A Particle Swarm Optimization-Based Multiuser Detection for Receive-Diversity-Aided. IEEE Signal Process. Lett..

[20] Zhang J., Chen S., Mu X., Hanzo L. (2014). Evolutionary-Algorithm-Assisted Joint Channel Estimation and Turbo Multiuser Detection/Decoding for OFDM/SDMA. IEEE Trans. Veh. Technol..

[21] Lazarescu M.T. (2013). Design of a WSN Platform for Long-Term Environmental Monitoring for IoT Applications. IEEE J. Emerg. Sel. Top. Circuits Syst..

[22] Zhang Y., Sun L., Song H., Cao X. (2014). Ubiquitous WSN for Healthcare: Recent Advances and Future Prospects. IEEE Internet Things J..

[23] Yuan F., Zhan Y., Wang Y. (2014). Data Density Correlation Degree Clustering Method for Data Aggregation in WSN. IEEE Sens. J..

[24] Omri A., Hasna M.O., Letaief K.B. (2015). Inter-Relay Interference Management Schemes for Wireless Multi-User Decode-and-Forward Relay Networks. IEEE Trans. Wirel. Commun..

[25] Sun Z., Akyildiz I.F. (2010). Channel Modeling and Analysis for Wireless Networks in Underground Mines and Road Tunnels. IEEE Trans. Commun..

[26] Huang W.-J., Hong Y.P., Kuo C.-C.J. (2008). Relay-Assisted Decorrelating Multiuser Detector (RAD-MUD) for Cooperative CDMA Networks. IEEE J. Sel. Areas Commun..

[27] Peng R.-M., Han Y., Gao Q. (2014). Distributed Estimation Scheme Based on Cooperative Communication in Wireless Sensor Networks. Wirel. Sens. Syst..

[28] Sampaio L.D.H., Souza A.R.C.E., Abrao T., Jeszensky P.J.E. (2014). Game Theoretic Energy Efficiency Design in MC-CDMA Cooperative Networks. IEEE Sens. J..

[29] Zappone A., Buzzi S., Jorswieck E. (2011). Energy-Efficient Power Control and Receiver Design in Relay-Assisted DS/CDMA wireless networksvia game theory. IEEE Commun. Lett..

[30] Yang H., Zhang Y. (2013). Analysis of Supercapacitor Energy Loss for Power Management in Environmentally Powered Wireless Sensor Nodes. IEEE Trans. Power Electron..

[31] Hunter T., Nosratinia A. Distributed Protocols for User Cooperation in Multi-User Wireless Networks. Proceedings of the IEEE Global Telecommunications Conference.

[32] Yao W., Chen S., Hanzo L. (2011). Generalized MBER-Based Vector Precoding Design for Multiuser Transmission. IEEE Veh. Technol. Soc..

[33] Sun Z., Akyildiz I.F. (2008). Channel modeling of wireless networks in tunnel. IEEE Trans. Commun..

